# Mortality from Amyotrophic Lateral Sclerosis and Parkinson’s Disease Among Different Occupation Groups — United States, 1985–2011

**DOI:** 10.15585/mmwr.mm6627a2

**Published:** 2017-07-14

**Authors:** John D. Beard, Andrea L. Steege, Jun Ju, John Lu, Sara E. Luckhaupt, Mary K. Schubauer-Berigan

**Affiliations:** ^1^Epidemic Intelligence Service, CDC; ^2^Division of Surveillance, Hazard Evaluations and Field Studies, National Institute for Occupational Safety and Health, CDC.

Amyotrophic lateral sclerosis (ALS) and Parkinson’s disease, both progressive neurodegenerative diseases, affect >1 million Americans (*1*,*2*). Consistently reported risk factors for ALS include increasing age, male sex, and cigarette smoking (*1*); risk factors for Parkinson’s disease include increasing age, male sex, and pesticide exposure, whereas cigarette smoking and caffeine consumption are inversely associated (*2*). Relative to cancer or respiratory diseases, the role of occupation in neurologic diseases is much less studied and less well understood (*3*). CDC evaluated associations between usual occupation and ALS and Parkinson’s disease mortality using data from CDC’s National Institute for Occupational Safety and Health (NIOSH) National Occupational Mortality Surveillance (NOMS), a population-based surveillance system that includes approximately 12.1 million deaths from 30 U.S. states.* Associations were estimated using proportionate mortality ratios (PMRs), standardizing indirectly by age, sex, race, and calendar year to the standard population of all NOMS deaths with occupation information. Occupations associated with higher socioeconomic status (SES) had elevated ALS and Parkinson’s disease mortality. The shifts in the U.S. workforce toward older ages and higher SES occupations^†^ highlight the importance of understanding this finding, which will require studies with designs that provide evidence for causality, detailed exposure assessment, and adjustment for additional potential confounders.

NOMS is a collaborative effort among 30 participating U.S. states’ Vital Statistics Offices (hereafter “states”),^§^ CDC’s NIOSH and National Center for Health Statistics (NCHS), and previously NIH’s National Cancer Institute and the U.S. Census Bureau. All participating states, or NCHS under states’ direction, share selected data from their death certificates with NIOSH through data sharing agreements. NOMS contains data on 12,710,846 deaths that occurred during 1985–1999, 2003–2004, and 2007–2011 in 30 states, although the number of states that contributed data in any 1 year was 10–22 (participation varied, related to funding and other concerns). After excluding 247,443 (2%) deaths among persons with ages reported as <18 years or >120 years, and 334,629 (3%) deaths without occupation information, 12,128,774 (95%) remaining deaths were included in this analysis.

ALS and Parkinson’s disease deaths were identified using *International Classification of Diseases*,^¶^ *9th Revision* (ICD-9) codes until 1998 and *10th Revision* (ICD-10) codes thereafter. ALS deaths were defined as decedents with underlying or contributing cause of death codes 335.2 (ICD-9) or G12.2 (ICD-10) and Parkinson’s disease deaths as those with underlying or contributing cause of death codes 332 (ICD-9) or G20 (ICD-10). Usual occupation,** recorded on death certificates in a text field, was assigned a U.S. Census 1990 or 2000 occupation code.^††^ These were converted to 2000 codes using a crosswalk based on U.S. Census data.^§§^ Occupation codes were then grouped into 26 categories based on similar job duties and ordered roughly from high SES (e.g., management) to low SES (e.g., transportation and material moving) (Table 1).^¶¶^ Associations between the 26 categories and ALS and Parkinson’s disease mortality were estimated via PMRs, standardizing indirectly by age, sex, race, and calendar year (*4*)***; 95% confidence intervals (CIs) for PMRs were calculated using formulas based on Byar's approximation to the exact Poisson test (*5*).

**TABLE 1:** The 26 occupation categories* derived from Census 2000 occupation codes^†^

Occupation category	Census 2000 occupation codes
Management	001–003, 034–035, 041, 043
Business operations	013, 015, 050–073
Financial	012, 080–095
Computer and mathematical	011, 100–124
Architecture and engineering	030, 130–156
Life, physical, and social science	036, 160–196
Community and social services	042, 200–206
Legal	210–215
Education, training, and library	023, 220–255
Arts, design, entertainment, sports, and media	006, 260–296
Health care practitioners and technical	300–354
Health care support	360–365
Protective service	370–395
Food preparation and serving	031, 400–416
Building and grounds cleaning and maintenance	420–425
Personal care and service	032–033, 430–465
Sales	004–005, 470–496
Office and administrative support	010, 040, 500–593
Farming, fishing, and forestry	020–021, 600–613
Construction	022, 620–676
Extraction	680–694
Installation, maintenance, and repair	700–762
Production	014, 770–896
Transportation and material moving	016, 900, 903–904, 907–909, 911–975
Military specific	983–985
Nonpaid workers	901–902, 905–906, 910

* Categories modified from the IPUMS website, which orders categories roughly from high to low socioeconomic status: https://usa.ipums.org/usa/volii/occ2000.shtml.

^†^ Census 1990 occupation codes were converted to Census 2000 occupation codes using a crosswalk based on data in Table 2 of U.S. Census Bureau Technical Paper #65: https://www.census.gov/people/io/files/techpaper2000.pdf.

Because cause-specific PMRs are mutually dependent, a higher mortality proportion for one cause results in a lower mortality proportion for another cause (*4*). Occupational categories reflect job duties and SES; therefore, higher SES occupations might have higher (or lower) PMRs for ALS and Parkinson’s disease because deaths from other causes which might be related to SES might be lower (or higher) in these occupations. To test whether this limitation of using PMRs for analysis might explain results for ALS and Parkinson’s disease, a sensitivity analysis was conducted in which chronic disease of the endocardium^†††^ was used as a negative control outcome (i.e., an outcome not expected to be related to occupation or SES) (*6*). A PMR pattern for chronic disease of the endocardium similar to that of ALS and Parkinson’s disease would suggest that higher (or lower) PMRs for ALS and Parkinson’s disease are caused by deficits (or surpluses) in other causes of death. Deaths for this additional analysis were defined as decedents with underlying or contributing cause of death codes for chronic disease of the endocardium (424 [ICD-9] or I34–I38 [ICD-10]).

The analysis included 26,917 ALS deaths, 115,262 Parkinson’s disease deaths, and 158,618 chronic disease of the endocardium deaths (Table 2). In crude analyses, ALS decedents were younger and more likely to be male and white than were decedents from all causes, whereas Parkinson’s disease decedents were older and more likely to be male and white than were decedents from all causes. Deaths from chronic disease of the endocardium were older and more likely to be female and white than decedents from all causes (Table 2).

**TABLE 2:** Crude frequencies and percentages for characteristics of deaths from all-causes, ALS,* Parkinson’s disease,^†^ and chronic disease of the endocardium^§^ — National Occupational Mortality Surveillance, United States, 1985–1999, 2003–2004, and 2007–2011

Characteristic	No. (%) total deaths^¶^	No. (%) ALS deaths^¶^	No. (%) Parkinson’s disease deaths^¶^	No. (%) chronic disease of the endocardium deaths^¶^
**Total**	**12,128,774 (100)**	**26,917 (100)**	**115,262 (100)**	**158,618 (100)**
**Age group (yrs)**				
18–25	162,518 (1)	39 (<1)	4 (<1)	290 (<1)
26–30	119,777 (1)	66 (<1)	2 (<1)	390 (<1)
31–35	152,495 (1)	167 (1)	4 (<1)	610 (<1)
36–40	195,859 (2)	338 (1)	10 (<1)	959 (1)
41–45	258,111 (2)	671 (2)	34 (<1)	1,398 (1)
46–50	353,626 (3)	1,122 (4)	131 (<1)	2,073 (1)
51–55	476,610 (4)	1,708 (6)	295 (<1)	2,853 (2)
56–60	648,794 (5)	2,545 (9)	759 (1)	4,166 (3)
61–65	900,238 (7)	3,601 (13)	2,143 (2)	6,452 (4)
66–70	1,169,674 (10)	4,471 (17)	5,783 (5)	10,129 (6)
71–75	1,456,778 (12)	4,492 (17)	13,603 (12)	15,948 (10)
76–80	1,699,612 (14)	3,913 (15)	25,509 (22)	23,442 (15)
81–85	1,787,507 (15)	2,508 (9)	31,599 (27)	31,542 (20)
86–90	1,508,379 (12)	1,009 (4)	23,998 (21)	32,108 (20)
91–95	884,866 (7)	226 (1)	9,395 (8)	19,512 (12)
96–100	299,456 (2)	38 (<1)	1,828 (2)	5,953 (4)
101–105	50,196 (<1)	3 (<1)	155 (<1)	735 (<1)
105–120	4,278 (<1)	0 (0)	10 (<1)	58 (<1)
Median±IQR	76 ± 21	69 ± 16	82 ± 10	82 ± 14
**Sex**				
Male	6,072,802 (50)	14,314 (53)	65,477 (57)	68,075 (43)
Female	6,055,972 (50)	12,603 (47)	49,785 (43)	90,543 (57)
**Race**				
White	10,633,589 (88)	25,279 (94)	109,281 (95)	146,195 (92)
Black	1,293,267 (11)	1,245 (5)	3,823 (3)	9,637 (6)
Other	201,918 (2)	393 (1)	2,158 (2)	2,786 (2)

**Abbreviations:** ALS = amyotrophic lateral sclerosis; ICD = *International Classification of Diseases*; IQR = interquartile range.

* Identified as deaths with the following ICD codes for the underlying or contributing causes of death: *9th Revision*: 335.2, *10th Revision*: G12.2.

^†^ Identified as deaths with the following ICD codes for the underlying or contributing causes of death: *9th Revision*: 332, *10th Revision*: G20.

^§^ Identified as deaths with the following ICD codes for the underlying or contributing causes of death: *9th Revision*: 424, *10th Revision*: I34–I38.

^¶^ 334,629 (3%) deaths from all-causes, 551 (2%) deaths from ALS, 1,853 (2%) deaths from Parkinson’s disease, and 2,791 (2%) deaths from chronic disease of the endocardium were excluded from this analysis because they were missing occupation information.

In standardized analyses, among ALS decedents, the PMRs for 14 occupation categories were significantly above 1.00, and for four (computer and mathematical; architecture and engineering; legal; and education, training, and library) were ≥1.50 (Table 3). In contrast, PMRs were significantly below 1.00 for 10 occupation categories, and none had a PMR ≤0.67 (Table 3). Among Parkinson’s disease decedents, PMRs for 13 occupation categories were significantly above 1.00, and none had a PMR ≥1.50. In contrast, PMRs were significantly below 1.00 for 11 occupation categories, and one (extraction [e.g., mining or oil and gas drilling]) had a PMR ≤0.67. Among chronic disease of the endocardium decedents, the PMRs for nine occupation categories were significantly above 1.00, but the magnitudes were much less than those observed for ALS and Parkinson’s disease; the highest (1.15) was for the legal category. The PMRs for seven occupation categories were significantly below 1.00, but, again, the magnitudes were much less than those observed for ALS and Parkinson’s disease; the lowest PMR for chronic disease of the endocardium was 0.81 (extraction category).

**TABLE 3:** Usual occupation category and mortality from ALS,* Parkinson’s disease,^†^ and chronic disease of the endocardium^§^ — National Occupational Mortality Surveillance, United States, 1985–1999, 2003–2004, and 2007–2011.

Census 2000 occupation categories^¶^	Total	ALS			Parkinson’s disease			Chronic disease of the endocardium		
	Deaths**	Deaths**		Standardized^††^ PMR (95% CI^§§^)	Deaths**		Standardized^††^ PMR (95% CI^§§^)	Deaths**		Standardized^††^ PMR (95% CI^§§^)
	Observed (No.)	Observed (No.)	Expected (No.)		Observed (No.)	Expected (No.)		Observed (No.)	Expected (No.)	
**Total**	**12,128,774**	**26,917**	**—**	**—**	**115,262**	**—**	**—**	**158,618**	**—**	**—**
Management	315,750	1,201	865	1.39 (1.31–1.47)	5,103	4,402	1.16 (1.13–1.19)	5,567	4,919	1.13 (1.10–1.16)
Business operations	92,346	367	248	1.48 (1.33–1.64)	1,178	1,040	1.13 (1.07–1.20)	1,383	1,299	1.06 (1.01–1.12)
Financial	142,828	509	376	1.35 (1.24–1.48)	2,147	1,716	1.25 (1.20–1.31)	2,103	2,061	1.02 (0.98–1.07)
Computer and mathematical	33,962	189	114	1.66 (1.43–1.91)	346	265	1.31 (1.17–1.45)	407	371	1.10 (0.99–1.21)
Architecture and engineering	208,426	845	544	1.55 (1.45–1.66)	3,663	2,847	1.29 (1.25–1.33)	3,115	2,842	1.10 (1.06–1.14)
Life, physical, and social science	59,989	215	156	1.38 (1.20–1.57)	931	701	1.33 (1.24–1.42)	843	782	1.08 (1.01–1.15)
Community and social services	97,004	304	223	1.36 (1.21–1.53)	1,482	999	1.48 (1.41–1.56)	1,357	1,270	1.07 (1.01–1.13)
Legal	43,936	178	110	1.62 (1.39–1.87)	703	500	1.40 (1.30–1.51)	674	584	1.15 (1.07–1.25)
Education, training, and library	426,012	1,431	857	1.67 (1.58–1.76)	6,148	4,203	1.46 (1.43–1.50)	6,918	6,534	1.06 (1.03–1.08)
Arts, design, entertainment, sports, and media	111,895	383	280	1.37 (1.23–1.51)	1,252	1,102	1.14 (1.07–1.20)	1,457	1,455	1.00 (0.95–1.05)
Health care practitioners and technical	299,250	950	710	1.34 (1.25–1.43)	3,325	2,772	1.20 (1.16–1.24)	4,542	4,279	1.06 (1.03–1.09)
Health care support	133,029	270	322	0.84 (0.74–0.94)	863	902	0.96 (0.89–1.02)	1,653	1,681	0.98 (0.94–1.03)
Protective service	148,058	396	393	1.01 (0.91–1.11)	1,295	1,509	0.86 (0.81–0.91)	1,641	1,676	0.98 (0.93–1.03)
Food preparation and serving	348,863	610	799	0.76 (0.70–0.83)	2,585	2,896	0.89 (0.86–0.93)	4,541	4,620	0.98 (0.95–1.01)
Building and grounds cleaning and maintenance	456,452	687	881	0.78 (0.72–0.84)	2,884	3,277	0.88 (0.85–0.91)	4,611	4,902	0.94 (0.91–0.97)
Personal care and service	338,556	816	729	1.12 (1.04–1.20)	2,019	2,208	0.91 (0.88–0.96)	3,055	3,290	0.93 (0.90–0.96)
Sales	861,453	2,318	2,044	1.13 (1.09–1.18)	10,004	9,357	1.07 (1.05–1.09)	11,934	11,648	1.02 (1.01–1.04)
Office and administrative support	895,316	2,534	2,132	1.19 (1.14–1.24)	9,631	8,717	1.10 (1.08–1.13)	13,245	13,135	1.01 (0.99–1.03)
Farming, fishing, and forestry	515,654	773	898	0.86 (0.80–0.92)	5,867	6,090	0.96 (0.94–0.99)	6,278	6,267	1.00 (0.98–1.03)
Construction	769,246	1,491	1,879	0.79 (0.75–0.83)	6,148	7,432	0.83 (0.81–0.85)	7,562	8,166	0.93 (0.91–0.95)
Extraction	81,813	132	192	0.69 (0.58–0.82)	599	942	0.64 (0.59–0.69)	761	945	0.81 (0.75–0.87)
Installation, maintenance, and repair	342,080	777	876	0.89 (0.83–0.95)	3,279	3,677	0.89 (0.86–0.92)	3,714	3,914	0.95 (0.92–0.98)
Production	1,322,655	2,721	2,964	0.92 (0.88–0.95)	12,578	13,902	0.90 (0.89–0.92)	16,437	16,901	0.97 (0.96–0.99)
Transportation and material moving	890,931	1,563	2,099	0.74 (0.71–0.78)	6,562	7,972	0.82 (0.80–0.84)	9,008	9,479	0.95 (0.93–0.97)
Military specific	110,555	286	290	0.99 (0.87–1.11)	1,131	1,182	0.96 (0.90–1.01)	1,178	1,236	0.95 (0.90–1.01)
Nonpaid workers^¶¶^	3,082,715	4,971	5,935	0.84 (0.81–0.86)	23,539	24,653	0.95 (0.94–0.97)	44,634	44,364	1.01 (1.00–1.02)

**Abbreviations:** ALS = amyotrophic lateral sclerosis; CI = confidence interval; ICD = *International Classification of Diseases*; NOMS = National Occupational Mortality Surveillance; PMR = proportionate mortality ratio.

* Identified as deaths with the following ICD codes for the underlying or contributing causes of death: *9th Revision*: 335.2, *10th Revision*: G12.2.

^†^ Identified as deaths with the following ICD codes for the underlying or contributing causes of death: *9th Revision*: 332, *10th Revision*: G20.

^§^ Identified as deaths with the following ICD codes for the underlying or contributing causes of death: *9th Revision*: 424, *10th Revision*: I34–I38.

^¶^ Census 1990 occupation codes were converted to Census 2000 occupation codes using a crosswalk based on data in Table 2 of US Census Bureau Technical Paper #65: https://www.census.gov/people/io/files/techpaper2000.pdf.

** 334,629 (3%) deaths from all-causes, 551 (2%) deaths from ALS, 1,853 (2%) deaths from Parkinson’s disease, and 2,791 (2%) deaths from chronic disease of the endocardium were excluded from this analysis because they were missing occupation information.

^††^ Indirectly standardized to the standard population of all NOMS deaths with occupation information by age, sex, race (white, black, other), and calendar year (1985–1989, 1990–1994, 1995–1998, 1999 and 2003–2004, 2007–2011). Different age group (years) categories were used for ALS, Parkinson’s disease, and chronic disease of the endocardium because the age distributions for these outcomes were different and numbers were small in the tails of the age distributions for ALS and Parkinson’s disease. The age group (years) categories that were used for ALS were ≤30, 31–35, 36–40, 41–45, 46–50, 51–55, 56–60, 61–65, 66–70, 71–75, 76–80, 81–85, 86–90, >90. The age group (years) categories that were used for Parkinson’s disease were ≤50, 51–55, 56–60, 61–65, 66–70, 71–75, 76–80, 81–85, 86–90, 91–95, 96–100, >100. The age group (years) categories that were used for chronic disease of the endocardium were 18–25, 26–30, 31–35, 36–40, 41–45, 46–50, 51–55, 56–60, 61–65, 66–70, 71–75, 76–80, 81–85, 86–90, 91–95, 96–100, 101–105, >105.

^§§^ Calculated using formulas based on Byar's approximation to the exact Poisson test (http://www.iarc.fr/en/publications/pdfs-online/stat/sp82/).

^¶¶^ Includes housewife or homemaker (2,789,320; 90%), volunteer (1,936; <1%), student (46,221; 1%), retired (51,567; 2%), and none, never worked, patient, disabled, or inmate (193,671; 6%).

## Discussion

Most previous studies of occupation and ALS and Parkinson’s disease have focused on exposures to toxicants (e.g., pesticides, solvents, lead, welding fume, and electromagnetic fields) that occur more frequently in lower SES occupations (e.g., farming, construction, production, and military service) (*1*–*3*). This study, however, did not find positive associations between lower SES occupations and ALS and Parkinson’s disease mortality; rather, positive associations were identified between ALS and Parkinson’s disease mortality and higher SES occupations such as computer and mathematical; architecture and engineering; legal; and education, training, and library occupations. Understanding the reasons for this finding is important for a number of reasons. The burdens of ALS and Parkinson’s disease mortality could increase in the future because the U.S. workforce is increasing in age, and increasing age is a recognized risk factor for ALS and Parkinson’s disease (*1*,*2*). If the associations between higher SES occupations and ALS and Parkinson’s disease mortality are real, then the burdens of ALS and Parkinson’s disease mortality could also increase in the future because the U.S. workforce is increasing in the number and proportion of workers employed in higher SES occupations. Substantially elevated PMRs for respiratory disease and injury-related mortality among extraction workers might explain lower PMRs for ALS and Parkinson’s disease in that occupation.

The findings in this report are subject to at least six limitations. First, usual occupation and outcomes might have been misclassified. A 1990 study based on 1980 U.S. Census occupation codes and 15 occupation categories reported the agreement between occupation ascertained from death certificates and company records was only 58% (*7*). However, a recent study based on 2010 U.S. Census Standard Occupational Classification codes and 22 occupation categories found the concordance between self-reported usual and current occupation was good (κ = 0.763; 95% CI = 0.754, 0.772) (*8*). Second, although the sensitivity of death certificates for ascertaining ALS is high (85%) (*9*), it is lower for Parkinson’s disease (56%) (*10*), which suggests misclassification of Parkinson’s disease deaths was likely more prevalent than misclassification of ALS deaths. Third, the broad occupation categories used for this analysis aggregated workers who might have had substantially different working conditions, limiting interpretation of results. For example, if an insecticide were positively associated with Parkinson’s disease mortality, this analysis might not have found a positive association between farming, fishing, and forestry and Parkinson’s disease mortality because that occupation category includes farmers who both did and did not use the insecticide as well as fishing and forestry workers who likely never used it. Fourth, death certificates do not collect dates of employment or of diagnosis, but the progressive natures of ALS and Parkinson’s disease make it unlikely that much of decedents’ time employed in their usual occupations would have occurred after diagnosis. Therefore, reverse causality (i.e., that diagnoses of ALS or Parkinson’s disease would cause workers to switch their usual occupations) and misclassification of usual occupation is unlikely. Fifth, this study was unable to separate effects of occupation and SES on ALS and Parkinson’s disease mortality, and results might have been affected by unmeasured confounders such as cigarette smoking. Finally, there are recognized limitations of using PMRs for analysis (*4*). The negative control outcome analysis, however, suggests that these limitations did not meaningfully affect results for higher SES occupations. Strengths of this study include its large sample size; complete, representative, and population-based sample, and that PMRs were indirectly standardized by measured confounders.

This study identified higher ALS and Parkinson’s disease mortality among workers in higher SES occupations, but was unable to identify occupational or nonoccupational factors that might explain these findings. Future studies of workers in higher SES occupations are needed to assess the consistency of these findings and identify factors that might explain elevated ALS and Parkinson’s disease mortality, using study designs that provide evidence for causality (e.g., cohort or case-control), individual exposure data for specific agents or experiences, and occupation categories formed on the basis of exposure to specific agents or experiences and linked to job exposure matrices for exposures of interest. Adjusting for potential confounding by cigarette smoking and socioeconomic status, using incidence rather than mortality to ascertain outcomes, and incorporating information regarding the timing of exposures relative to the timing of outcomes might help further elucidate the reasons for these findings, so that strategies for prevention could be developed.

**Summary**

**What is already known about this topic?**

Amyotrophic lateral sclerosis (ALS) and Parkinson’s disease are progressive neurodegenerative diseases that affect >1 million Americans. Factors consistently reported to be either positively or inversely associated with ALS and Parkinson’s disease are primarily demographic or behavioral. The role of occupation in these diseases is relatively understudied and poorly understood.

**What is added by this report?**

This study described the burden of ALS and Parkinson’s disease mortality by usual occupation in a large, complete, representative, and population-based sample in the United States and found higher ALS and Parkinson’s disease mortality among workers in occupations associated with higher socioeconomic status (SES).

**What are the implications for public health practice?**

Although the reasons for the findings of this study are not understood, it provides information for future targeted studies among workers in higher SES occupations to identify risk factors for ALS and Parkinson’s disease. These studies should use designs that provide evidence for causality, detailed exposure assessment, and adjustment for additional potential confounders.

## Footnotes

* https://www.cdc.gov/niosh/topics/NOMS/.

† The shifts in the U.S. workforce mentioned can be seen by comparing tables of data from the Bureau of Labor Statistics’ Current Population Survey for the years 2011 and 2015 at https://www.bls.gov/cps/demographics.htm.

§ Data for this study were provided by Vital Statistics Offices from the following U.S. states: Alaska, Colorado, Georgia, Hawaii, Idaho, Indiana, Kansas, Kentucky, Louisiana, Maine, Michigan, Missouri, Nebraska, Nevada, New Hampshire, New Jersey, New Mexico, North Carolina, North Dakota, Ohio, Oklahoma, Rhode Island, South Carolina, Tennessee, Texas, Utah, Vermont, Washington, West Virginia, and Wisconsin.

¶ https://www.cdc.gov/nchs/icd/.

** Usual occupation was ascertained on death certificates via the following field: DECEDENT’S USUAL OCCUPATION (Indicate type of work done during most of working life. DO NOT USE RETIRED). The standard U.S. death certificate is available at https://www.cdc.gov/nchs//nvss/mortality_methods.htm.

†† U.S. Census 1990 and 2000 occupation codes are available at https://www.census.gov/people/io/methodology/.

§§ The crosswalk is based on data in Table 2 of U.S. Census Bureau Technical Paper #65: https://www.census.gov/people/io/files/techpaper2000.pdf.

¶¶ Bureau of Labor Statistics tables showing occupation by educational attainment and occupation by income are available at https://www.bls.gov/emp/ep_table_111.htm and https://www.bls.gov/oes/current/oes_nat.htm, respectively.

*** For example, the PMR for ALS for the management category was calculated as the observed number of ALS deaths in the management category divided by the expected number of ALS deaths in that category. The expected number of ALS deaths for management was calculated as the sum of the stratum-specific expected numbers of ALS deaths for management, where the strata were defined by crosstabulations of the variables used for standardization. The stratum-specific expected numbers of ALS deaths in the management category were calculated as the stratum-specific observed numbers of ALS deaths for all occupation categories multiplied by the stratum-specific observed numbers of deaths from all causes in the management category divided by the stratum-specific observed numbers of deaths from all causes for all occupation categories.

††† Chronic disease of the endocardium includes nonrheumatic mitral valve disorders; nonrheumatic aortic valve disorders; nonrheumatic tricuspid valve disorders; pulmonary valve disorders; and endocarditis, valve unspecified.
